# Supplementary material for: Responses to the Tepotinib in Gastric Cancers with MET Amplification or MET Exon 14 Skipping Mutations and High Expression of Both PD-L1 and CD44
Source: Cancers (Basel). 2022 Jul 15;14(14):3444. doi: 10.3390/cancers14143444 (PMC9318186; doi:10.3390/cancers14143444)
Supplement: Supplementary file 1 [file cancers-14-03444-s001.zip › Supplementary materials_Figure S1 and S2.pptx]

## Slide 1
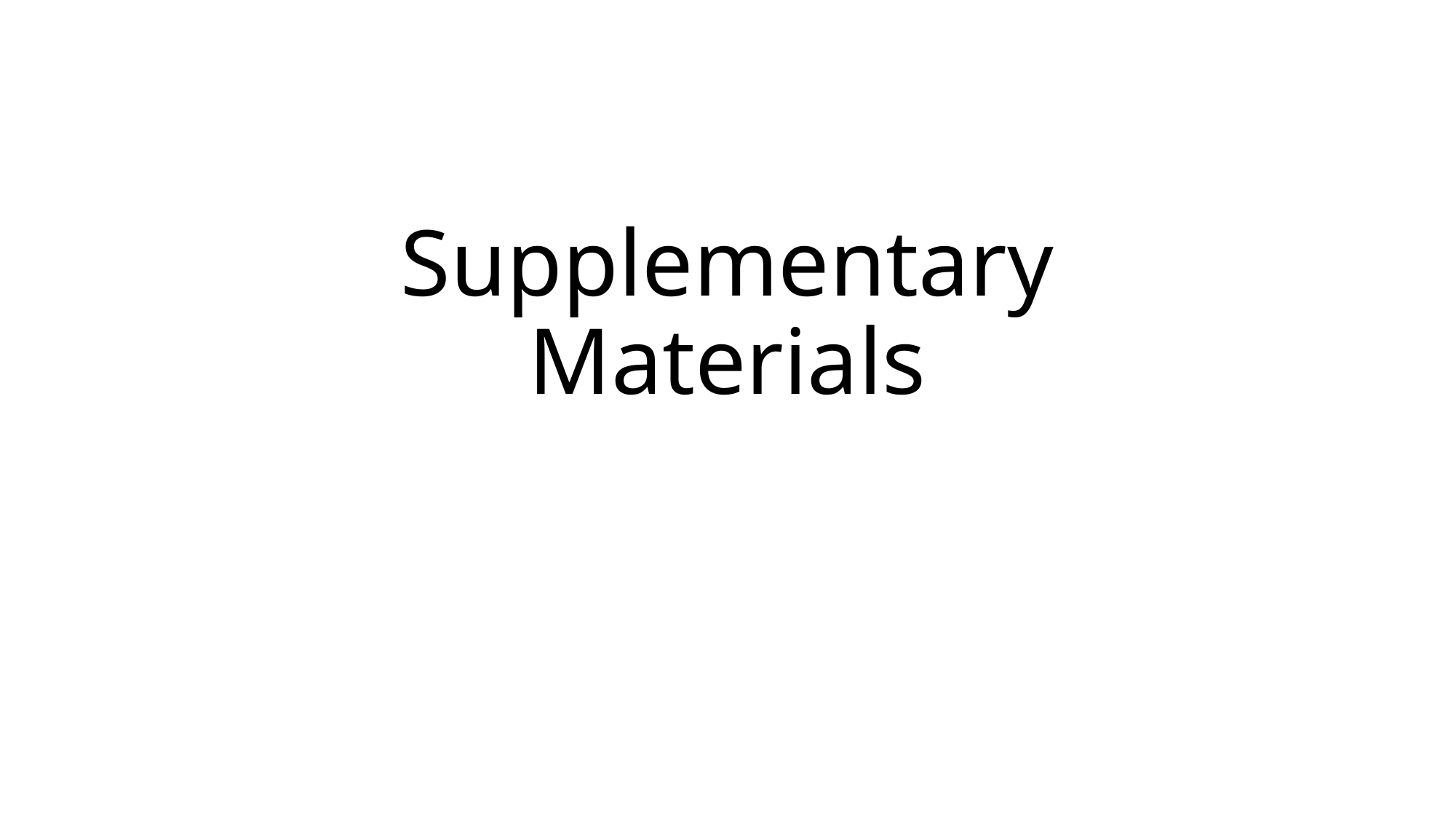

# Supplementary Materials

## Slide 2
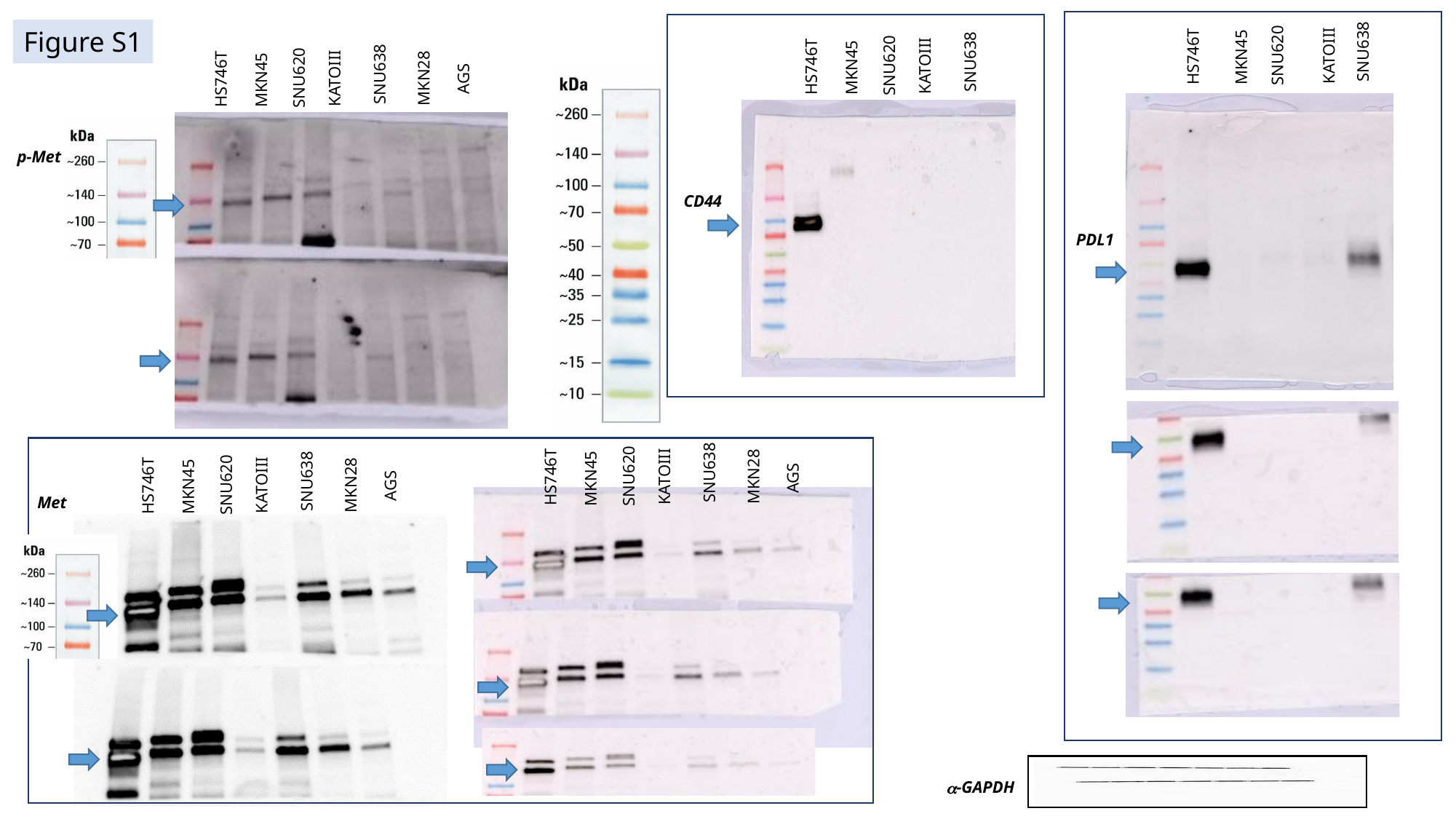

SNU638
KATOIII
MKN45
SNU620
 HS746T
SNU638
KATOIII
MKN45
SNU620
 HS746T
Figure S1
SNU638
AGS
MKN28
KATOIII
MKN45
SNU620
 HS746T
CD44
p-Met
PDL1
SNU638
AGS
MKN28
KATOIII
MKN45
SNU620
 HS746T
SNU638
AGS
MKN28
KATOIII
MKN45
SNU620
 HS746T
Met
a-GAPDH

## Slide 3
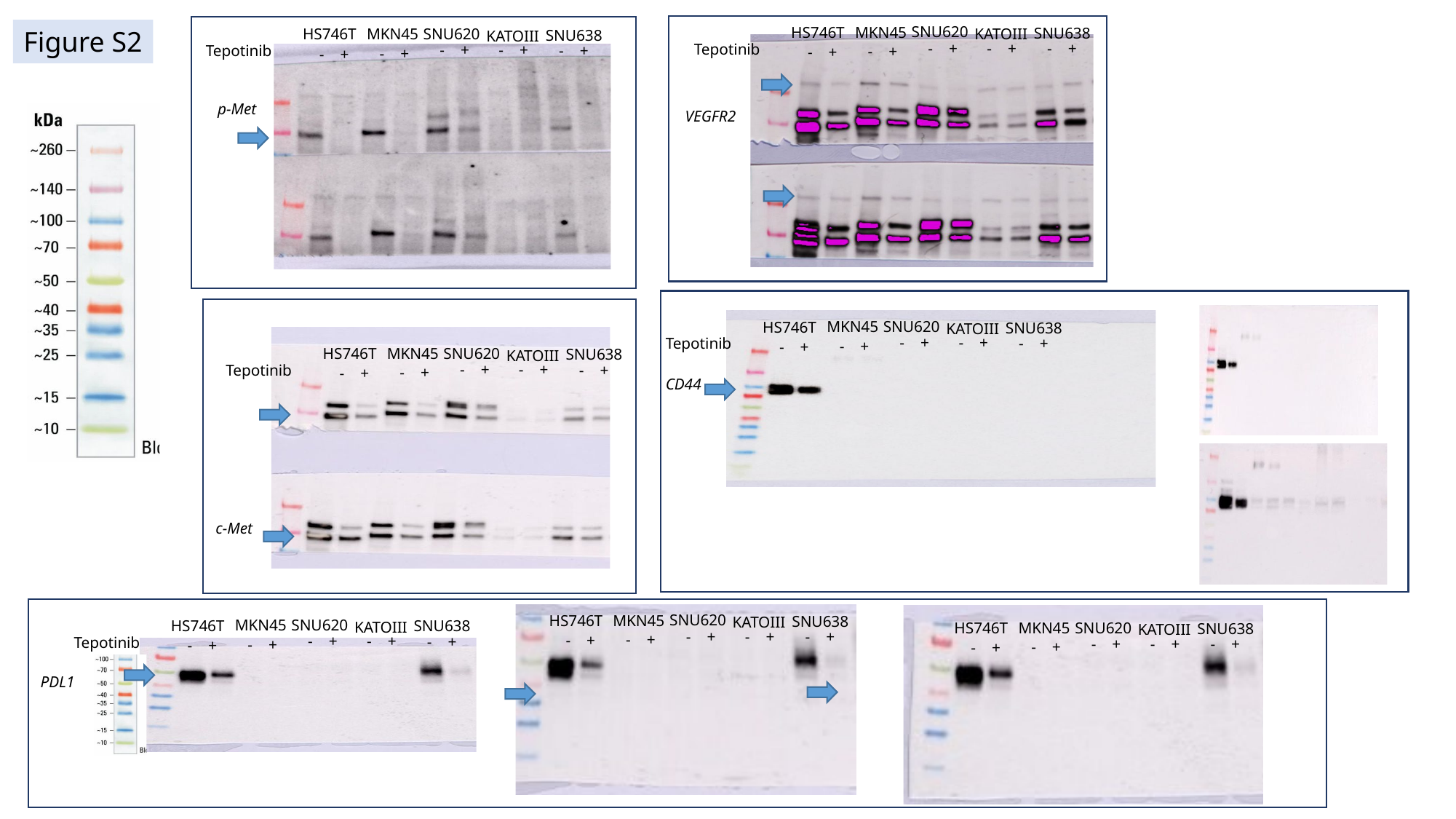

SNU620
MKN45
HS746T
SNU638
KATOIII
- +
- +
- +
Tepotinib
- +
- +
SNU620
MKN45
HS746T
SNU638
KATOIII
- +
- +
- +
Tepotinib
- +
- +
Figure S2
VEGFR2
p-Met
CD44
SNU620
MKN45
HS746T
SNU638
KATOIII
- +
- +
- +
Tepotinib
- +
- +
c-Met
SNU620
MKN45
HS746T
SNU638
KATOIII
- +
- +
- +
Tepotinib
- +
- +
SNU620
MKN45
HS746T
SNU638
KATOIII
- +
- +
- +
- +
- +
SNU620
MKN45
HS746T
SNU638
KATOIII
- +
- +
- +
Tepotinib
- +
- +
SNU620
MKN45
HS746T
SNU638
KATOIII
- +
- +
- +
- +
- +
PDL1

## Slide 4
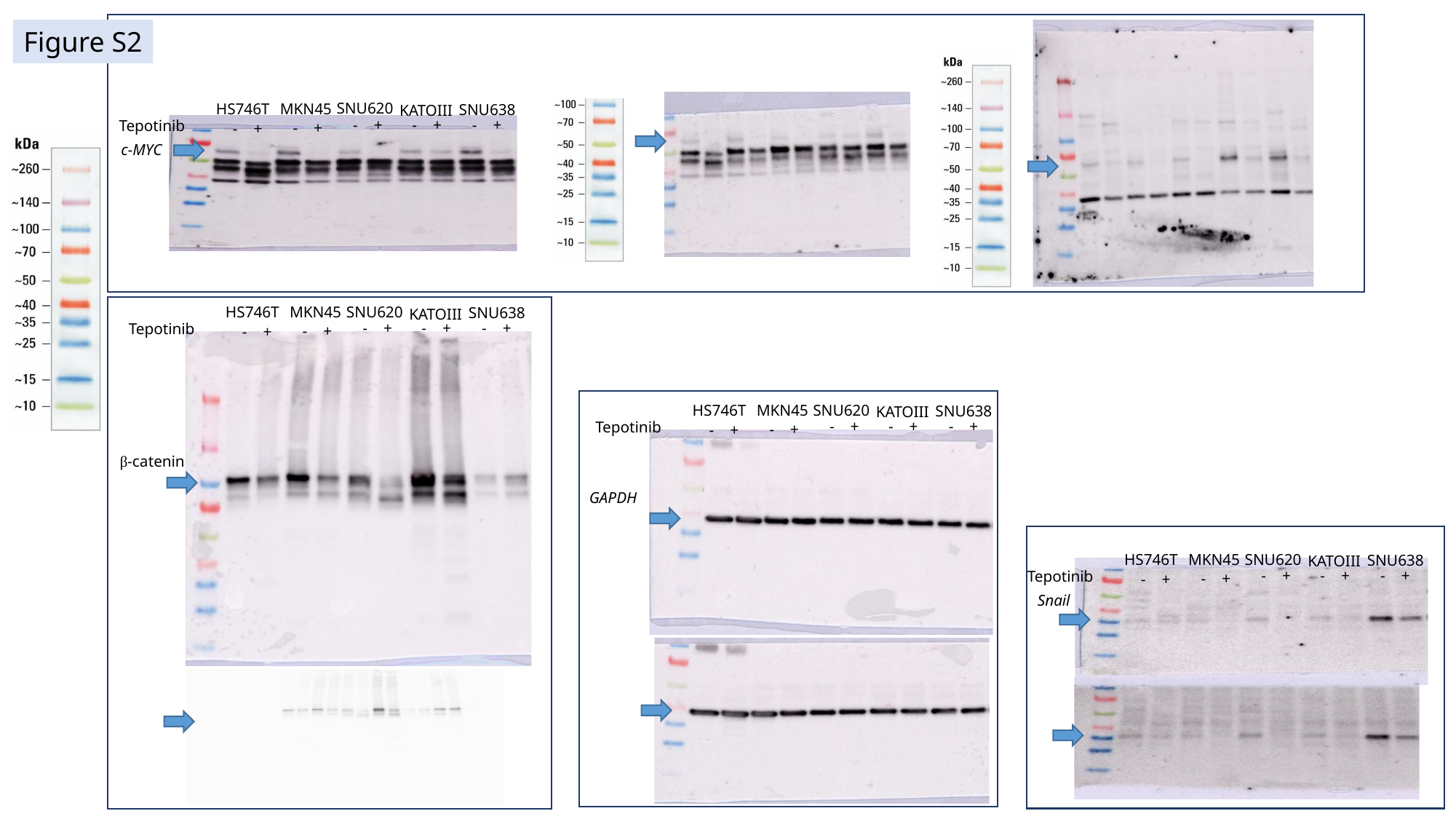

Figure S2
SNU620
MKN45
HS746T
SNU638
KATOIII
- +
- +
- +
Tepotinib
- +
- +
c-MYC
SNU620
MKN45
HS746T
SNU638
KATOIII
- +
- +
- +
Tepotinib
- +
- +
SNU620
MKN45
HS746T
SNU638
KATOIII
- +
- +
- +
Tepotinib
- +
- +
β-catenin
GAPDH
SNU620
MKN45
HS746T
SNU638
KATOIII
- +
- +
- +
Tepotinib
- +
- +
Snail
